# Bitter Taste Receptors and Endocrine Disruptors: Cellular and Molecular Insights from an In Vitro Model of Human Granulosa Cells

**DOI:** 10.3390/ijms232415540

**Published:** 2022-12-08

**Authors:** Francesca Paola Luongo, Sofia Passaponti, Alesandro Haxhiu, Maryam Raeispour, Giuseppe Belmonte, Laura Governini, Livio Casarini, Paola Piomboni, Alice Luddi

**Affiliations:** 1 Department of Molecular and Developmental Medicine, Siena University, 53100 Siena, Italy; 2 Department of Life Sciences, University of Siena, 53100 Siena, Italy; 3 Unit of Endocrinology, Department of Biomedical, Metabolic and Neural Sciences, University of Modena and Reggio Emilia, 41126 Modena, Italy

**Keywords:** endocrine disruptors, taste receptors, hGL5, steroidogenesis, mitochondria, lipid droplets

## Abstract

Endocrine disrupting chemicals (EDCs) are compounds that interfere with the synthesis, transport and binding action of hormones responsible for reproduction and homeostasis. Some EDCs compounds are activators of Taste bitter Receptors, a subclass of taste receptors expressed in many extraoral locations, including sperm and follicular somatic cells. This makes TAS2Rs attractive molecules to study and investigate to shed light on the effect of EDCs on female reproduction and fertility. This study aims to assess the effect of selected EDCs [namely Biochanin A (BCA), caffeine, Daidzein, Genistein and Isoflavone] on hGL5, an immortalized cell line exhibiting characteristics coherent with primary follicular granulosa cells. After demonstrating that this model expresses all the TAS2Rs (TAS2R3, TAS2R4, TAS2R14, TAS2R19, TAS2R43) specifically expressed by the primary human granulosa cells, we demonstrated that BCA and caffeine significantly affect mitochondrial footprint and intracellular lipid content, indicating their contribution in steroidogenesis. Our results showed that bitter taste receptors may be involved in steroidogenesis, thus suggesting an appealing mechanism by which these compounds affect the female reproductive system.

## 1. Introduction

Lifestyle and environmental factors can adversely affect fertility. Exposure to Endocrine Disrupting Chemicals (EDCs) affects both male and female fertility [1,2]. EDCs are defined as compounds that interfere with the synthesis, transport, metabolism or binding action of hormones responsible for reproduction, homeostasis and developmental processes [3]. Endocrine disruptor molecules are highly heterogeneous and include synthetic chemicals used as industrial solvents/lubricants and their byproducts, plastics, plasticizers, pesticides, fungicides, and pharmaceutical agents and some natural chemicals present in human and animal food, such as phytoestrogens. Genistein can also act as an endocrine disruptor and is widely consumed [4]. These compounds are structurally similar to hormones and exert action via non-nuclear steroid hormone receptors, nuclear receptors, non-steroid receptors, orphan receptors and steroid synthesis enzymatic pathways or other pathways involved in the endocrine and reproductive system. The concern over the presumed effects of various chemicals released into the environment on the reproduction of humans and other species is increasing. EDCs are also associated with epigenetics changes and reprogramming [5,6]; they can methylate DNA and target the cytosine residues located in cytosine-phosphate-guanine (CpG) dinucleotides [7]. Histone modification often occurs concomitantly with DNA methylation and results in short- and long-term alterations in transcription program; another endocrine epigenetic alteration is the aberrant expression of microRNAs (miRNAs). The miRNAs are closely related to small interfering RNAs (siRNAs) that are involved in DNA methylation and histone modifications [8]. A growing number of studies are investigating the effects of EDCs, particularly BPA, on the reproductive system have been conducted in recent decades [8], including using granulosa in animal models [9,10] and in humans [11]. There is increasing evidence from previous studies [12,13] demonstrating the role of EDCs in the pathogenesis of some female reproductive disorders, including polycystic ovarian syndrome, premature ovarian failure, endometriosis, reproductive tract anomalies, uterine fibroids and ectopic gestation.

EDCs are reported to interact with Bitter Taste Receptors (TAS2Rs) [14], a subclass of taste receptors that specialize in perceiving bitter compounds (such as toxic plant alkaloids). Although it was traditionally thought that taste receptors are expressed exclusively on the taste buds of the tongue, recent evidence has shown that this family of receptor mediates non-gustatory functions in other parts of the body [15]. In fact, taste receptors are expressed in many extraoral locations, such as the liver [16], respiratory system [15], pancreas [2], urinary bladder [17], brain, ovaries [18], testes [19,20,21] and sperm [20,21,22].

TAS2Rs are G protein-coupled receptors with seven transmembrane domains, encoded by more than 25 genes [23]. A recent study reported the expression of TAS2R3, TAS2R4, TAS2R14, TAS2R19 and TAS2R43 in human follicular somatic cells, both Granulosa cells (GCs) and Cumulus Cells (CCs), and the presence of these TAS2Rs was previously demonstrated in the testes and in male gametes, which is representative of the importance of TAS2R2 in the human reproductive system [22,23]. To the best of our knowledge, there are no studies investigating the possible relationship between EDCs, taste receptors and infertility. The latter condition, defined by World Health Organization (WHO) as the inability to conceive after 1 year of unprotected intercourse, affects about 15% of couples of reproductive age [24]. Female fertility mainly relies on the oocyte quality and competence, which, in turn, are the result of a complex and dynamic developmental relationship between the developing oocyte and the neighboring follicular somatic cells [25]. Indeed, the oocyte induces the proliferation of GCs; in turn, these cells are able to provide the oocyte with signals and molecules that directly affect its differentiation. Therefore, GCs represent an attractive tool to non-invasively study the possible molecular mechanisms underlying the effects of EDCs on female fertility. However, the small number of primary GCs collected during the oocyte retrieval has been shown to be inadequate for many gene regulatory studies; moreover, the genetic architecture of the population may be a bias for interpreting the results. In this study, we propose the use of immortalized human granulosa cell line (hGL5), a cell line that is derived from primary luteinized GC cells after transformation with the E6 and E7, open reading frames, regions of human papillomavirus androstenedione 16 [26]. hGL5 represents a useful tool for basic research and clinical application on GCs, including studies on gonadotropin-dependent proliferation, cell survival and apoptosis [27]. These cells are an attractive model for investigating the mechanisms relating to steroid biosynthesis and other pathways involved in GC metabolic functions. We therefore exposed hGL5 to selected EDCs reported to be agonists of TAS2Rs. To provide insights into their activity on these ovarian cells, we measured mitochondrial morphology, lipid droplets amount and steroid hormones secretion.

## 2. Results

### 2.1. hGL5 Express Bitter Taste Receptors

**Figure 1:**
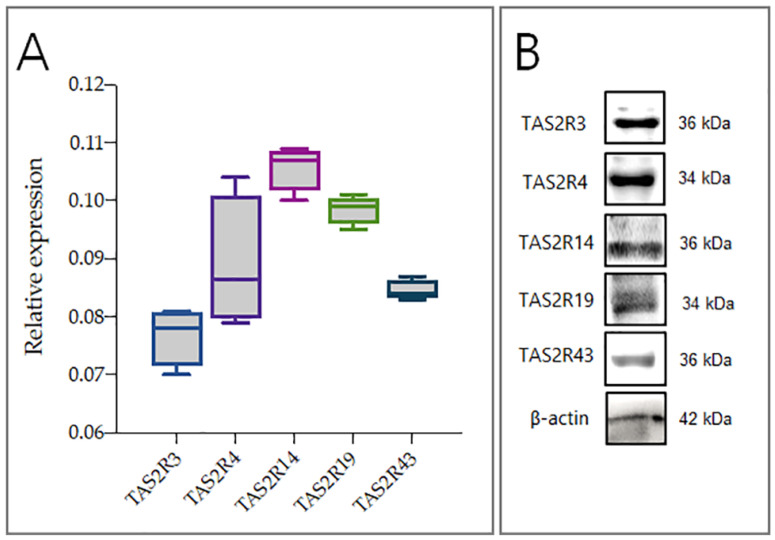
Expression of TAS2Rs in hGL5. (Panel **A**) TAS2R3, TAS2R4, TAS2R14, TAS219, TAS2R43 relative expression in hGL5. Graphical diagrams are plotted as box–whisker plots, where boxes show the interquartile range with median and mean values. (Panel **B**) Representative image of western blot analysis of TAS2Rs in hGL5. Equal protein loading was verified using the housekeeping ß-Actin. Image representative of three independent experiments.

hGL5 exhibit characteristics consistent with primary follicular granulosa cells, including cell retraction in response to activation of protein kinase-A; the ability to produce progesterone and estradiol; and expression of P450 aromatase, the enzyme involved into the estradiol production [28]. All of these features make hGL5 a suitable model for studying the effects of EDC on steroid biosynthesis. Therefore, to clarify the molecular pathways underlying the activity of TAS2Rs in granulosa cells, hGL5 were employed. As shown in Figure 1A, hGL5 have TAS2Rs, with TAS2R14 being the most expressed (*p* < 0.05). The intracellular levels of mRNA were confirmed at protein level; indeed, western blot analysis revealed the presence of all investigated proteins, with different relative abundance (Figure 1B).

### 2.2. Effect of EDCs Exposure on Viability of hGL5 Cells

**Figure 2:**
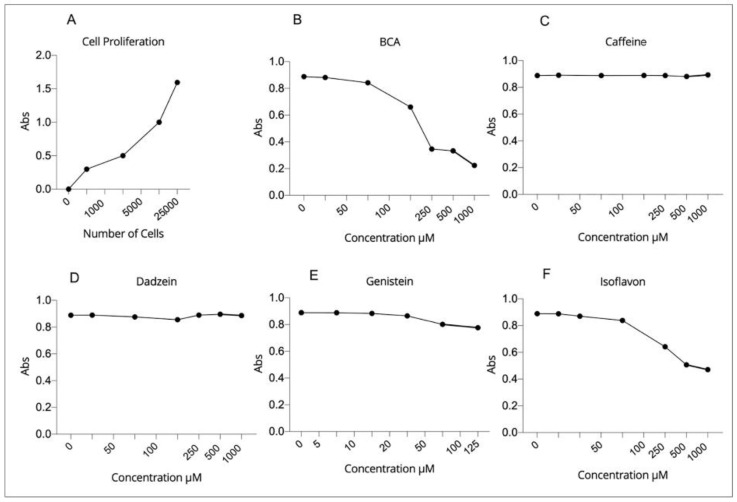
(**A**) Cell proliferation assay for establishing the number of cells to use for further tests. (**B**–**F**) Cytotoxicity assay for evaluation of the toxicity effects of bitter compounds on hGL5 cells, using cell counting kit-8 (Abcam). The nontoxic concentration for each compound was calculated: 10 µM for BCA (**B**), 300 µM for caffeine (**C**), 500 µM for daidzein (**D**), 5 µM for genistein (**E**) and 25 µM for isoflavone (**F**).

The cell proliferation test was performed to determine the number of cells for the following treatments (Figure 2A). Next, we analyzed the viability of hGL5 cells after exposure to selected EDCs, which are reported to be specific agonists of bitter taste receptors. hGL5 remains highly proliferating, providing an optimal tool for the cell toxicity assay, which requires a robust number of cells to be performed. According to literature, all the compounds were tested up to the maximum concentration of 1000 µM, except genistein, which was tested at the highest concentration of 250 µM. Biochanin A (BCA) showed a toxic effect on hGL5 cells at concentrations above 20 µM (Figure 2B) and isoflavone at a concentration higher than 50 µM (Figure 2F). For caffeine (300 µM), daidzein (500 µM) and genistein (5 µM), no toxic effects were observed, confirming results described in other studies [14,29] (Figure 2C–E).

Based on these results, the non-toxic concentration of each compound on hGL5 cells was as follows: BCA: 20 µM; Caffeine: 300 µM; Daidzein: 500 µM; Genistein: 5 µM; Isoflavone: 50 µM.

### 2.3. EDCs Acts as Agonists on TAS2Rs Affecting Mitochondrial Footprint

**Figure 3:**
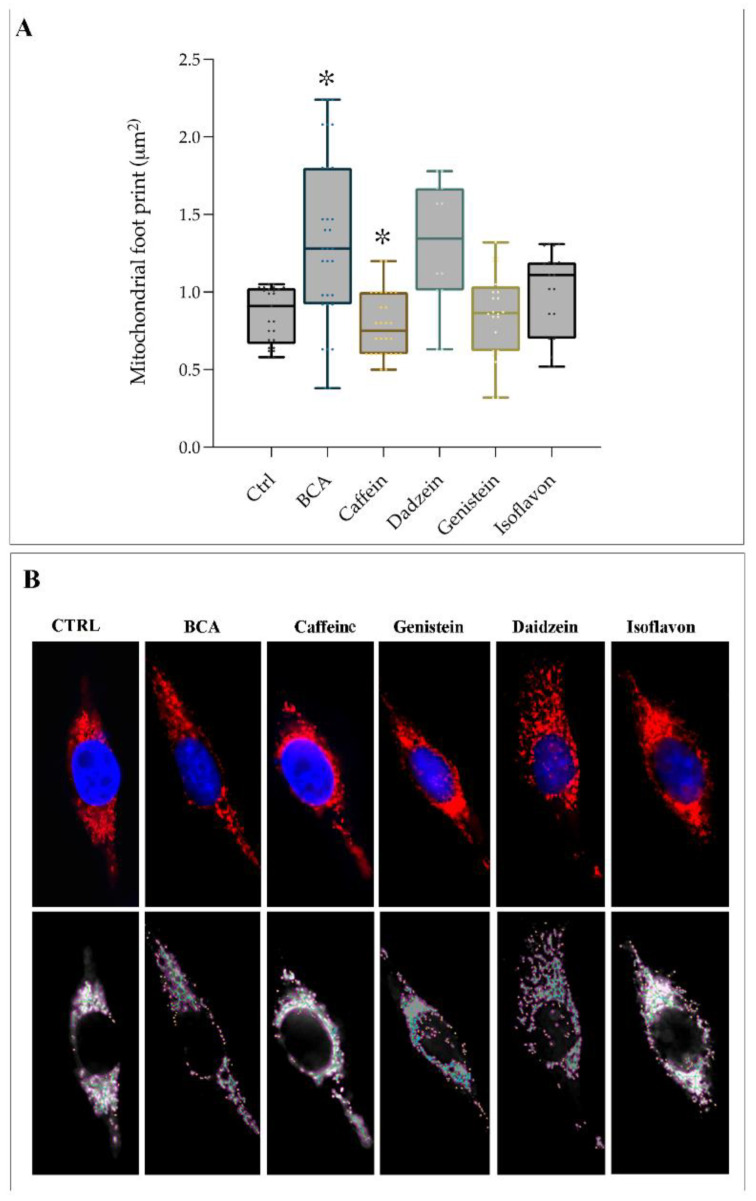
Results from mitochondrial network analysis in hGL5. (**A**) Summary statistics for all cells. Box plots show median (horizontal lines), first to third quartile (box), and the most extreme values within 1.5 times the interquartile range (vertical lines). Differences between control and TAS2Rs agonists-treated cells were statistically evaluated. * *p* < 0.05). (**B**) Mitochondrial footprint (red) of cells exposed to different compounds. Nuclei were DAPI-stained (blue). CTRL = untreated cell (control). Magnification: 630×. Staining pictures are representative of five independent experiments.

In 2017, Shi Pan et al. reported that TAS2R agonists (chloroquine and quinine) induce human airway smooth muscle (ASM) cell death by affecting mitochondria through modulating their structure and function [30]. Mitochondrial morphology mirrors the health of the cell. To better characterize mitochondrial shape and dynamics in stimulated hGL5 cells, we took advantage of the fluorescent dye MitoTracker, which uses the mitochondrial membrane potential to label mitochondria within living cells. The stained cells were then analyzed by Mitochondrial Network Analysis (MiNA). This toolset, which enables semiautomated analysis of mitochondrial morphologies [31], gains a better understanding of mitochondrial dynamics as it relates to cell health. This software skeletonized the MitoTracker Red images and produced an outputs collection of mitochondrial descriptors, such as the number of individuals and networks, mean branch size, mean branch length and network size. Therefore, using the MiNA macros, we were able to observe the dynamic nature of the mitochondria in hGL5 treated with TAS2Rs agonists. As shown in Figure 3, BCA significantly increased the mitochondrial footprint and the median length of branches (*p* < 0.05). In contrast, hGL5 treated with caffeine showed mitochondrial compaction, loss of branches and an obvious reorganization of the mitochondria distribution. Indeed, as shown in Figure 3A, caffeine significantly reduced the mitochondrial footprint (*p* < 0.05), the area occupied by mitochondrial structures, and the number of branches per network, suggesting mitochondrial fragmentation. No significant modification occurred in mitochondria of hGL5 treated with other compounds.

**Figure 4:**
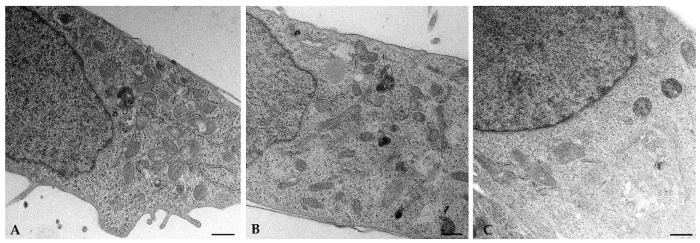
Representative transmission electron microscopy (TEM) images of untreated (**A**), 24 h BCA- (**B**) or caffeine-treated hGL5 (**C**). Mitochondria of cells treated with BCA are elongated and tubular when compared to the round mitochondria found in control cells, whereas mitochondria of cells treated with caffeine are smaller and rounded. Scale bar: (**A**,**C**), 500 nm; (**B**), 200 nm. Image representative of three independent experiments.

Electron microscopy confirmed the morphological changes we observed by fluorescence probing. In cells stimulated by BCA, the mitochondria have a larger diameter and appear elongated and tubular compared to the round mitochondria present in the control cells (Figure 4). Interestingly, ultrastructure showed that large portions of filamentous ER appeared near enlarged mitochondria in steroid-producing cells. Smaller and round mitochondria are present in hGL5 treated with caffeine (Figure 4C), according to results from mitochondrial footprint analysis (Figure 3).

### 2.4. BCA and Caffeine Affect TAS2Rs Relative Abundance

**Figure 5:**
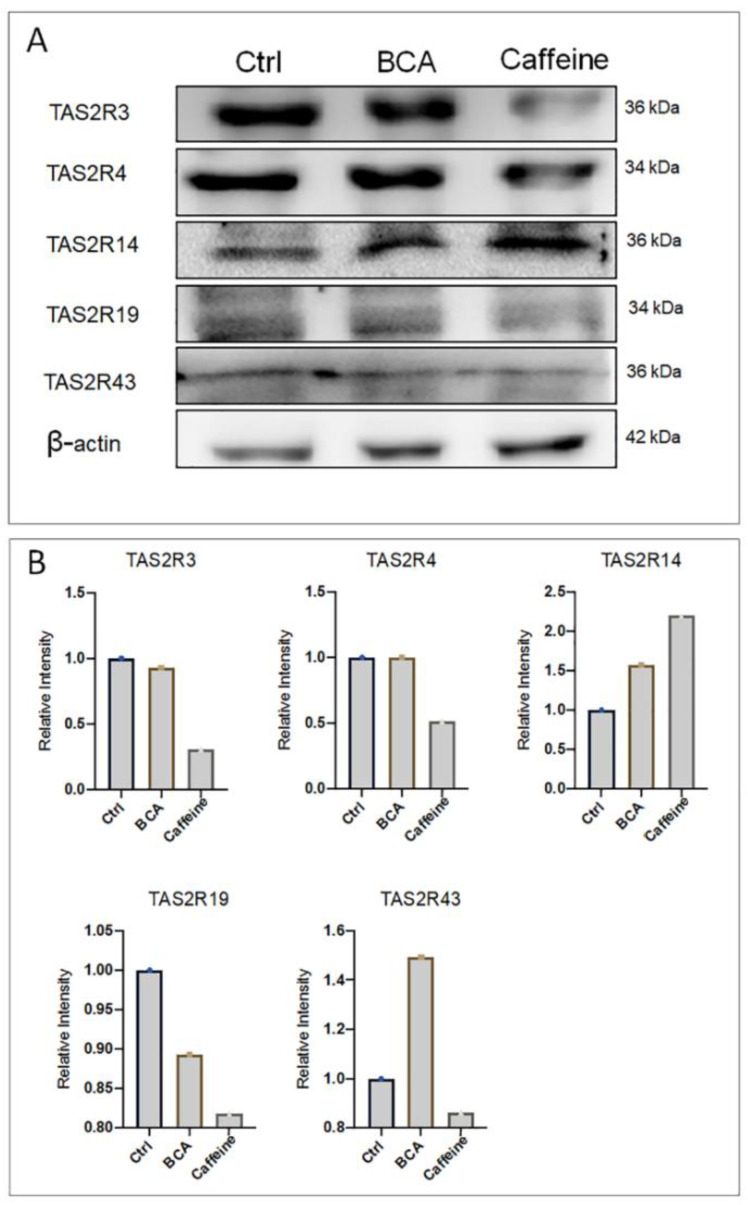
TAS2Rs protein expression in the hGL5 cell line. (**A**) Representative images of three independent western blot analysis of TAS2Rs after treatment with BCA and caffeine. β-actin was used as a loading control. (**B**) Relative quantification of western blotting band intensities.

Based on previous experiments that showed BCA and caffeine to be the most effective agonists of TAS2Rs, we evaluated the relative abundance of each TAS2Rs after treatment with agonists. As shown in Figure 5, BCA (10 µM) increased the expression of TAS2R14 and TAS2R43 and decreased the relative abundance of TAS2R19. Caffeine (300 µM) significantly decreased the relative abundance of TAS2R3, TAS2R19 and TAS2R43, but increased the relative abundance of TAS2R14.

### 2.5. BCA and Caffeine Affect Intracellular Lipid Storage and Steroid Secretion

**Figure 6:**
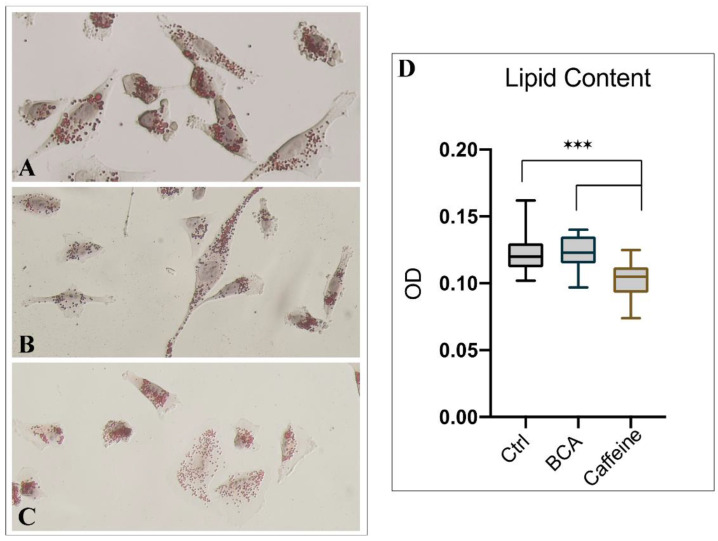
Lipid droplets analysis. Morphological characteristics of lipid droplets in cultured hGL5 cells. (**A**) Untreated samples. (**B**) 24 h, BCA-treated cells. (**C**) Caffeine-treated cells. Magnification: 400×. (**D**) Lipid content quantification by Oil red O extraction. *** Ctrl vs. Caffein and BCA vs. Caffein: significance *p* < 0.0001.

A close relationship between taste receptors and steroid hormones has been demonstrated thus far. Therefore, to assess whether the effect of BCA and caffeine (two recognized agonists of TAS2Rs) on mitochondria may be a steroidogenic input, we evaluated the shape and the load of hGL5 lipid droplets. These latter are indeed of functional importance in steroidogenesis, as they store cholesterol substrate for the synthesis of steroid hormone. Changes in the morphology of lipid droplets in hGL5 treated with caffeine and BCA were evaluated using Oil red O staining and are shown in Figure 6A. Morphological changes in lipid droplets manifested by fusing with each other, and its size changed after fusion. In hGL5 treated with caffeine, smaller lipid droplets may be observed, which were generally spherical in shape (Figure 6B). In contrast, BCA-treated hGL5 tended to form relatively larger lipid droplets, which were shown to be spherical or irregular (Figure 6C). Intracellular lipids within hGL5 were also measured by Oil red O extraction. As shown in Figure 6D, we found that lower amounts of lipids were deposited in hGL5 cells after 24 h of in vitro culture with caffeine. Additionally, after Oil red O staining, the color of lipid droplets was generally brighter in the control group compared with BCA and caffeine. Because the color intensity reflected the levels of triglycerides and cholesterol within lipid droplets, these data demonstrated that higher lipid levels were accumulated in the control group in comparison to the treated group. Tukey’s Multiple Comparison test shows that lipid content was reduced significantly when comparing caffeine to the control and BCA (*p* < 0.0001).

**Figure 7:**
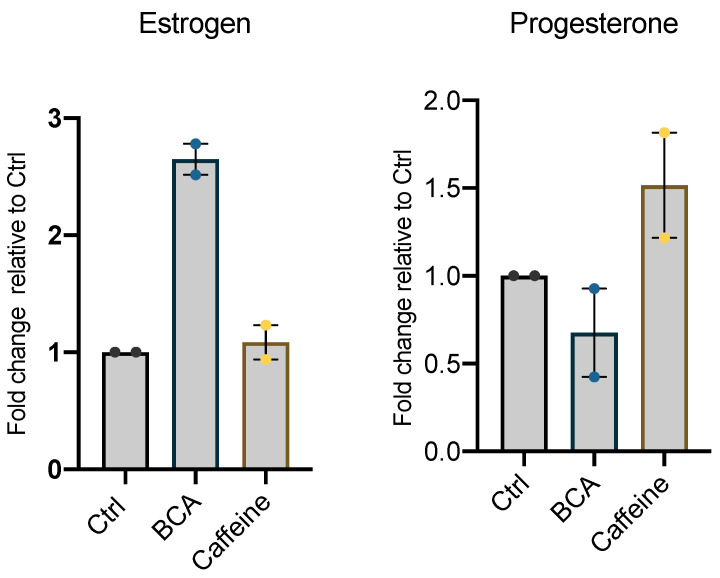
The effect of BCA and caffeine on P_4_ secretion from hGL5. The cells were cultured for 24 h with BCA 50 μM or caffeine. Data are expressed as a fold change relative to control culture (set as 1).

Finally, we measured the secretion of steroid hormones (estrogen and progesterone) in the culture medium of untreated hGL5 or treated with BCA and caffeine for 24 h. As shown in Figure 7, BCA induced an increase in E2 secretion (*p* < 0.05), while caffeine did not; conversely, BCA induced a decrease, although not statistically significant, in P4 secretion.

## 3. Discussion

In this study, we have mainly revealed the expression of different taste receptors in hGL5, an immortalized cell line exhibiting features coherent with primary follicular granulosa cells. We also provided evidence of significant modification of the mitochondrial footprint by BCA and caffeine, two agonists of taste receptors, which are also able to modulate the expression of TAS2Rs in hGL5 cells. Finally, we reported morphological changes in lipid droplets and a significant decrease in the amount of lipids deposited in hGL5 cells after 24 h of in vitro culture with caffeine.

Endocrine disruptors can affect the endocrine system without interacting directly with receptors, interfering with the synthesis or metabolism of steroid hormones.

The consequences of exposure to EDCs and the mechanism of action is dependent on some factors, including the age of exposure, latency from exposure, the dose of the exposure and transgenerational and epigenetic effect [32]. A survey of more than 30 isoflavones and structurally related compounds reported that BCA, daidzein and genistein inhibit key enzymes biosynthesis of neurosteroid and/or steroid hormones [33]; however, another study demonstrated the dose-dependent effect of the isoflavones on steroidogenesis, in which, at the lowest dose, the process is inhibited, while positive responses were shown at the highest dose [34].

These confounding factors should be limited by in vitro studies on well-standardized cellular models. In this respect, the use of primary granulosa cells may present limitations, such as difficulties in harvesting and handling, short-term maintenance in culture, lack of availability in large numbers and missing responsiveness after collection to gonadotropins due to the receptor downregulation [35]. Moreover, they do not proliferate in vitro. Therefore, the human ovarian cell line hGL5 have been proposed to be an appealing alternative in vitro model [36]. Here, we confirm the efficacy of hGL5 cells by demonstrating that they express bitter taste receptors, which we have previously reported to be expressed in primary granulosa cells [37]. Notably, as previously seen for primary granulosa cells, in hGL5, TAS2R14 is also the most expressed taste GPCR.

TAS2R14 is specifically activated by resveratrol [15,23,38], a natural polyphenol, which, at the ovarian level, is capable of protecting oocytes by reducing oxidative stress [39], increasing the total number of oocytes and decreasing the apoptotic index of GC. A huge amount of data supports the estrogenic activity of resveratrol and its potential role as EDC. In this regard, we have shown that the treatment of hGL5 with selected EDCs and caffeine, reported as agonists of these receptors, modulates TAS2R relative abundance at a lower concentration than those used to activate TAS2Rs in HEK293 cells [39].

We have shown that cellular treatment with BCA induces a significant increase in relative abundance of TAS2R14, as well as in mitochondrial footprint and in length of mitochondrial branches. Mitochondria are the central sites for the biosynthesis of steroid hormones [40], and mitochondrial dynamic changes, namely fission and fusion, are a key feature of steroidogenic cells [41]. Indeed, mitochondria have been shown to be key factors controlling female reproductive processes. Previous studies demonstrated that the biosynthesis of steroid hormones in steroidogenic cells is closely associated with mitochondrial dynamic changes, thus supporting that cellular and molecular changes in mitochondria would affect steroidogenesis [41]. Duarte and colleagues demonstrated that mitochondrial fusion is involved in the regulation of steroid synthesis [42]. Indeed, mitochondrial reorganization and contact between membranes represents the first step in steroid synthesis and secretion through the plasma membrane. Note that mitochondrial fusion (associated with increased mitochondrial footprint) is observed during the production of steroid hormones, while a reduction occurs in mitochondrial fission [43]. Moreover, a higher complexity of mitochondrial *cristae* has been reported after exposure of rat granulosa cells to LH [44]. We demonstrated that caffeine significantly decreased the mitochondrial footprint and the mean of the branches’ length, thus suggesting mitochondrial fragmentation. Our data, together with the direct correlation found in primary human cumulus cells between serum estradiol levels, mitochondrial mass and mitochondrial membrane potential [40], suggest the existence of an effect of BCA and caffeine in steroid metabolism. In this regard, we showed that treatment with BCA induces estrogen secretion while reducing P4 secretion. These data support previous studies reporting a dose-dependent effect of different isoflavones. In fact, Nynca et al. [45] concluded that biochanin A and other isoflavones inhibit P4 secretion, while their effect on E2 production depends on the phytoestrogen examined and follicular maturity.

We also reported a significant modulation in the amount and size of lipid droplets after cell treatment with BCA and caffeine. The increased amount of lipids after exposure to BCA is consistent with increased mitochondrial footprint. An increase in intracellular lipid content has been reported alongside the follicle growth, with an exponential rise in large antral follicles [46] and in cultured granulosa cells [47]. This can reflect the different capabilities of GCs to respond to hormonal signals and provide energy for follicle development. In light of this evidence, increased mitochondrial footprint and lipid droplets induced by BCA treatment suggests the recruitment of lipids into droplets during the differentiation of granulosa cells into hormone-responsive steroidogenic cells.

## 4. Materials and Methods

### 4.1. Study Design

The objective of this study was to assess the effect of endocrine disruptors on hGL5, a human ovarian cell line, and evaluate the expression of Taste receptor type 2.

### 4.2. hGL5 Cell Line

hGL5 cells were cultured in Dulbecco Modified Eagle Medium (Invitrogen, Whaltman, MA, USA) containing fetal bovine serum (10%), L-glutamine (1%), penicillin/streptomycin (1%), non-essential amino acids (1%) and Ultra serum G (Sartorius, Goettingen, Germany) (2%). Cultures were maintained in the incubator (5% CO_2_ at 37 °C).

### 4.3. Cell Proliferation

Cell proliferation assay was performed using cell counting kit-8 CCk-8 (Abcam, Cambridge, UK). Serial dilution starting from 25,000 hGL5 cells were plated in 96 wells and incubated overnight. 10 µL of CCk-8 solution was added gently to the wells, avoiding air bubbles. After incubating the plate for 4 h at 37 °C (5% CO_2_), the absorbance was measured at 450 nm using a microplate reader, according to the manufacturer’s instructions. For each concentration, 8 well counts were measured, and the average data were used to prepare the calibration curve.

### 4.4. Compounds

We employed 5 compounds, previously described as activators of TAS2Rs and EDCs in the literature. Genistein ≥98%, Daidzein ≥98%, Biochanin A and caffeine purchased from Sigma Aldrich (St. Louis, MO, USA) and 3′,4′,7-Trihydroxyisoflavone (Santa Cruz Biotechnology, Santa Cruz, CA, USA) were dissolved in dimethyl sulfoxide (DMSO), ethanol, acetone and H_2_O, respectively, and diluted in the media containing Dulbecco’s Modified Eagle Medium (DMEM, 1X), containing fetal bovine serum (FBS) (10%), L-glutamine (1%), penicillin/streptomycin (1%), non-essential amino acids (1%) and Ultra serum (2%), not exceeding a final DMSO concentration of 0.1% (*v/v*) to avoid the toxic effect of the cells. For acetone and ethanol, the toxicity effect was measured separately.

### 4.5. Cytotoxicity Assay

To investigate the effect of Genistein, Daidzein, 3′,4′,7-Trihydroxyisoflavone, BCA and caffeine of the viability of the hGL5 cells, cytotoxicity assay using cell counting kit-8 were conducted after overnight incubation at 37 °C (5% CO_2_), with each compound at different concentrations with serial dilution in consideration of the optimal concentration to stimulate TAS2Rs previously reported in the literature. The high doses of these compounds were chosen based on the short time of exposure (for only 24 h). The absorbance was measured after 4 h incubation (37 °C, 5% CO_2_) with CCK-8 (10 µL). For each compound, 4 well counts were carried out, and the average was calculated. For the control of background absorbance, the absorbance of wells containing media without cells was measured, and the average was subtracted from other wells. The wells with cells but without stimulation was also prepared as a control group for the cytotoxicity assay.

### 4.6. Mitotracker

The hGL5 cells were plated over cover slip and after 24 h of treatment with compounds (BCA, Caffeine, Dadzein, Genistein, Isoflavon); they were incubated at 37 °C for 15 min with MitoTracker^®^ Red CMXRos probes, which diffuse across the plasma membrane and accumulate in active mitochondria. After incubation, slides were fixed with Methanol at −20 °C for 15 min and followed by 3 times washing with PBS 1X and with mounting and visualization.

### 4.7. RNA Extraction Complementary DNA Preparation

Total RNA was isolated from hGL5 cells with RNeasy Protect Mini kit, according to the manufacturer’s instructions (Qiagen, Hilden, Germany), as previously reported [48]. At the end of the protocol, RNA extracted from each sample was diluted in a final volume of 30 Μl of water. The purity and the concentration of RNA were evaluated by reading on NanoDrop^®^ ND-100 UV-vis Spectrophotometer (Thermo Fisher Scientific, Waltham, MA, USA). 1 µg of the extracted RNA was reverse transcribed into cDNA, using the iScript Gdna Clear TM cDNA Synthesis Kit (Bio-Rad Laboratories, Hercules, CA, USA). We started with a treatment of samples with a master mix of DNase to remove contaminating genomic DNA. To inactivate the effect of DNase, the samples were incubated in a thermal cycler at 25 °C for 5 min and at 75 °C for another 5 min. 4 µL of the iScript reverse Transcription Supermix was added to each sample; they were then incubated in a thermal cycler (5 min at 25 °C, 20 min at 46 °C, 1 min at 95 °C).

### 4.8. qPCR

Gene expression was evaluated using a specific EvaGreen assay, annealing only on the exon-exon junction sequence of the specific mRNA, ensuring selective amplification of the target genes, thus excluding genomic DNA contamination. To normalize the expression levels of the gene transcripts hGL5, a simultaneous mRNA expression profiling of the housekeeping gene *HPRT1* was also performed in all the analyzed samples. All amplification reactions were conducted in triplicate by qRT-PCR on a CFX connect Real-Time PCR Detection System (Bio-Rad Laboratories) using SsoFast EvaGreen Supermix (Bio-Rad Laboratories); gene-specific primer sets used in this study are listed in Table S1. Melting curve analysis was also performed to confirm the specificity of the products obtained. Changes in gene expression levels were calculated by the 2^−ΔΔCt^ method.

### 4.9. Western Blot Analysis

Western blotting was performed as previously described; 50 μg of total protein were diluted in Laemmli buffer, as previously described [49], kept at 95 °C for 5 min and separated on 10% polyacrylamide gel using the Cell Mini Protean (Bio-Rad). After electrophoresis, the gel was transferred onto a nitrocellulose membrane (GE Healthcare, Chicago, IL, USA) in a Mini Trans-Blot apparatus (Bio-Rad). The gel was then blocked for 1 h in 5% nonfat dry milk and then incubated overnight at 4 °C with primary antibodies (see Table S2) diluted in 1% nonfat dry milk/TTBS (TBS containing 0.2% Tween 20). After washing in TTBS, the membrane was incubated with the appropriate horseradish peroxidase (HRP)-conjugated secondary antibody (see Table S2). The same nitrocellulose was also incubated with an anti-β-actin antibody (Microscience, Wokingham, UK), followed by the secondary antibody (Table S2), as an internal loading control. Immunostained bands were visualized by chemiluminescence with ImageQuant LAS 4000 (GE Healthcare).

### 4.10. Oil Red O (ORO) Staining and Morphological Observation

The amount of lipids accumulated in hGL5 cell line was evaluated using the Oil red O staining method according to a previously described protocol [50], with slight modifications. 0.5 g of ORO were resuspended in 100 mL of isopropanol (ORO stock solution). The working solution was prepared with 30mL of this stock diluted with 20 mL ddH_2_O (ORO-saturated solution). Following EDC treatments, cells were washed 3 times with PBS and fixed with 4% paraformaldehyde for 10 min. Cells were then washed twice with PBS, and were subsequently stained with Oil red O (Sigma Aldrich) in the dark for 15 min at room temperature. Thereafter, cells were stained with hematoxylin, washed with PBS and photographed using a phase contrast microscope (Olympus, Tokyo, Japan). For the quantification of the droplets, cells were plated in 96 wells, following the aforementioned protocol with a difference in the final step with the addition of isopropanol to extract Oil red O. Supernatant was subjected to determination of optical density (OD) value at 540 nm using the automatic enzyme immunoassay analyzer.

### 4.11. Transmission Electron Microscopy

Cells were fixed in 2.5% glutaraldehyde as previously described [51] and observed with a FEI Tecnai G2 Spirit transmission electron microscope (Hillsboro, OR, USA).

#### ELISA Test

The secretion of P_4_ and E_2_ from hGL5 was measured in the culture medium of hGL5 treated for 24 h with BCA and caffeine. The concentration of steroid hormones in spent culture medium was measured by Immulite 2000 (Siemens).

### 4.12. Statistical Analysis

Statistical analysis was performed with GraphPad Prism 9.0 (GraphPad Software, San Diego, CA, USA). All data obtained from the experiments were analyzed for normality using the Shapiro-Wilk or Kolmogorov-Smirnov tests, followed by the parametric or non-parametric tests, ANOVA or Kruskal-Wallis respectively, as appropriate. Densitometric analyses were completed using ImageJ software. Statistical significance was set at *p* < 0.05.

## 5. Conclusions

In summary, these results confirm the hGL5 cell line as a suitable model to study the effect of EDCs in vitro follicular cells. In addition, the correlation of the distribution and amount of lipid droplets after treatments provide new evidence that EDCs may regulate steroidogenesis through TAS2Rs.

## Data Availability

Not applicable.

## Supplementary Materials

The following supporting information can be downloaded at: https://www.mdpi.com/article/10.3390/ijms232415540/s1.
