# Supplementary material for: Bitter Taste Receptors and Endocrine Disruptors: Cellular and Molecular Insights from an In Vitro Model of Human Granulosa Cells
Source: Int J Mol Sci. 2022 Dec 8;23(24):15540. doi: 10.3390/ijms232415540 (PMC9779643; doi:10.3390/ijms232415540)
Supplement: Supplementary file 1 [file ijms-23-15540-s001.zip › Supplementary table .pdf]

Table S1: **PrimePCR Gene Expression Assays.**

Target gene	Acronym	Assay ID
Taste receptor, type 2, member 3	*TAS2R3*	dHsaEG5003946
Taste receptor, type 2, member 4	*TAS2R4*	dHsaEG5003947
Taste receptor, type 2, member 14	*TAS2R14*	dHsaEG5004113
Taste receptor, type 2, member 19	*TAS2R19*	dHsaEG5003736
Taste receptor, type 2, member 43	*TAS2R43*	dHsaEG5004567
**Reference gene**	**Acronym**	**Assay ID**
Hypoxanthine-Guanine Phosphoribosyl Transferase	*HPRT1*	dHsaEG5189658

Table S2 : List of Antibodies used in this study.

Antigen	*Donor Species*	*Dilution*	*Manufacter*	*RRID*
Primary antibodies				
TAS2R3	Rabbit	1:500	Thermo Fisher Scientific	AB_2556263
TAS2R4	Rabbit	1:500	Thermo Fisher Scientific	AB_2201090
TAS2R14	Rabbit	1:500	NovusBio Laboratories	AB_11053160
TAS2R19	Rabbit	1:1000	Thermo Fisher Scientific	AB_962285
TAS2R43	Rabbit	1:1000	Thermo Fisher Scientific	AB_2815963
B actin	Mouse	1:2000	Bio-Rad laboratories	AB_2223350
Secondary antibodies				
Anti-Mouse-IgG HRP	Goat	1:8000	Bio-Rad laboratories	AB_609692
Anti-Rabbit-IgG HRP	Goat	1:8000	Bio-Rad laboratories	AB_1102634
